# Fabrication of uniform 4H-SiC mesopores by pulsed electrochemical etching

**DOI:** 10.1186/1556-276X-9-570

**Published:** 2014-10-13

**Authors:** Jia-Hui Tan, Zhi-zhan Chen, Wu-Yue Lu, Yue Cheng, Hong He, Yi-Hong Liu, Yu-Jun Sun, Gao-Jie Zhao

**Affiliations:** 1 Department of Physics, Shanghai Normal University, 100 Guilin Road, Shanghai 200234, China

**Keywords:** Constant pulsed current, Uniform mesopores, Cycle time, Pause time

## Abstract

In this letter, the uniform 4H silicon carbide (SiC) mesopores was fabricated by pulsed electrochemical etching method. The length of the mesopores is about 19 μm with a diameter of about 19 nm. The introduction of pause time (*T*_off_) is crucial to form the uniform 4H-SiC mesopores. The pore diameter will not change if etching goes with *T*_off_. The hole concentration decreasing at the pore tips during the *T*_off_ is the main reason for uniformity.

## Background

Silicon carbide (SiC) is a promising semiconductor for high-temperature, high-power, and high-frequency electronic devices, due to its wide bandgap, high breakdown electric field, and high electron saturation velocity [1]. Recently, the advent of high surface-to-volume ratio of SiC porous semiconductors has drawn significant attention. SiC porous structures are found applications in the gas sensors [2], supercapacitor [3], and RF planar inductor [4]. Cao et al. [5] fabricated a porous layer in amorphous SiC thin films by using constant-current anodic etching in an electrolyte of aqueous diluted HF. The morphology of the porous layer was dependent on the anodic current density. Ke et al. [6] fabricated variable porous structures on 6H-SiC crystalline faces by anodic etching under constant voltage with UV assistance. The nano-columnar pore was formed on the C face. With simplified experimental conditions, Gautier et al*.*[7] formed columnar mesopores on 4H-SiC under constant-current density without UV assistance. The pore size was not uniform. It was about 10 nm near the surface and between 20 and 50 nm at a 40-μm depth when the current density was 25.5 mA/cm^2^.

Uniform pore structures can enhance the optical performance and can be applied in optoelectronics field [8]. In this paper, the uniform longitudinal distribution of SiC mesopores on 4H-SiC substrate with thin cap and transition layer was first fabricated by pulsed electrochemical etching. The uniform pore diameter of about 19 nm, in the whole 4H-SiC mesopores, was prepared through the introduction of pause time.

## Methods

**Figure 1:**
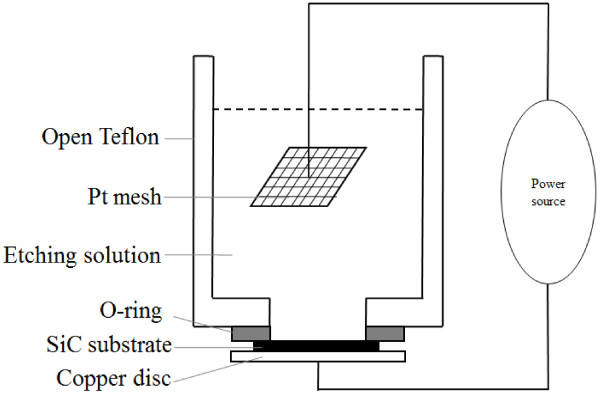
The schematic drawing of electrochemical etching.

The 4H-SiC mesopores were fabricated on the C face of a n-type 4H-SiC wafer with on-axis surface. The wafer is 340-μm thick and double polished and the resistivity is about 0.015 ~ 0.028 Ω · cm. All samples were prepared in the size of 1 cm × 1 cm. The C face was chosen as an etching face. Samples 1, 2, 3, 4, and 7 (#1, #2, #3, #4, #7) were made to be Schottky contacts by sticking a 0.2-mm-thick copper foil with conductive silver glue. A 200-nm-thick nickel was sputtered on the Si face of samples 5 and 6 (#5, #6), and then annealed in N_2_ atmosphere for 3 min to form an Ohmic contact. The Keithley 2635A semiconductor characterization system (Keithley, Cleveland, USA) was used to acquire I-V curves to determine whether the backside contact is Ohmic or Schottky. The schematic drawing of anodic etching system is shown in Figure 1. The anodization was performed in a simple, open Teflon cell with an O-ring seal (201 mm^2^ active area) under constant room temperature. A copper disc, with a copper wire, being pressed to the metal contact side of the sample, served as a conductor in order to integrate the sample in the anodic etching system. Pt mesh works as a counter electrode. The etching solution was used with volume ratios HF (49%):C_2_H_5_OH (99%):H_2_O_2_ (30%) of 3:6:1. Etching time of all samples was 7 min. All experiments were processed under room light. After anodization, they were rinsed and then cracked along <1000 > axis. The morphology of the cross sections were observed by HITACHI S-4800 scanned electron microscopy (SEM) (Hitachi High-Technologies Corporation, Tokyo, Japan).

The pulsed current cycle time and the pause time are represented by “T” and “T_off_,” respectively. The current pulse shapes from the power source are measured by oscilloscope. It is proved to be approximately rectangular in shape in longer *T* (10 ms) and triangular in shape in shorter *T* (0.2 ms). To modulate the *T* and *T*_off_, the constant pulsed current/voltage resource is applied. The etching was performed in galvanostatic mode for samples #1 to #6 and in potentiostatic mode for sample #7. The detailed experimental parameters are listed in Table 1.

**Table 1:** Experimental parameters

**Sample number**	**Contact property**	**Etching mode**	***T *** **(ms)**	***T***_**off **_**(ms)**
#1	Schottky contact	Constant pulsed current J = 35 mA/cm^2^	10	0
#2	Schottky contact	Constant pulsed current J = 35 mA/cm^2^	10	5
#3	Schottky contact	Constant pulsed current J = 35 mA/cm^2^	0.4	0
#4	Schottky contact	Constant pulsed current J = 35 mA/cm^2^	0.4	0.2
#5	Ohmic contact	Constant pulsed current J = 50 mA/cm^2^	0.4	0
#6	Ohmic contact	Constant pulsed current J = 50 mA/cm^2^	0.4	0.2
#7	Schottky contact	Constant voltage (14 V) with pulsed current	0.4	0

## Results and discussion

The porous layer structure consists of two parts, one is the cap and transition layer, the other is the mesopores. The characteristics of different porous layer structure were summarized in Table 2. The thickness of cap and transition layer is about 1 μm, and the length of mesopores is about 19 μm for all samples. The mesopore diameters are 19 nm in the middle and more than 30 nm in the bottom for samples #1, #3, #5, and #7. They are always about 19 nm from the middle to the bottom for samples #2, #4, and #6. Tolerances of all parameters are shown in Table 2. Here, we select about 50 mesopores in different parts of a sample and get an average value of pore diameter and tolerances. The mesopore wall thickness is almost the same in different cycle times and pause times and gets to be thinner when the current densities increase to a higher value [5-7].

**Table 2:** The characteristics of porous layer structure

**Sample number**	**Cap and transition layer (μm)**	**Characteristics of mesopores**		
		**Length of mesopores (μm)**	**Mesopore diameter in the middle (nm)**	**Mesopore diameter in the bottom (nm)**
#1	1 (±0.3)	19 (±0.5)	19 (±5)	33 (±5)
#2	1 (±0.3)	19 (±0.5)	19 (±5)	19 (±5)
#3	1 (±0.3)	19 (±0.5)	19 (±5)	33 (±5)
#4	1 (±0.3)	19 (±0.5)	19 (±5)	19 (±5)
#5	1 (±0.3)	19 (±0.5)	19 (±5)	38 (±5)
#6	1 (±0.3)	19 (±0.5)	19 (±5)	19 (±5)
#7	1 (±0.3)	19 (±0.5)	19 (±5)	33 (±5)

**Figure 2:**
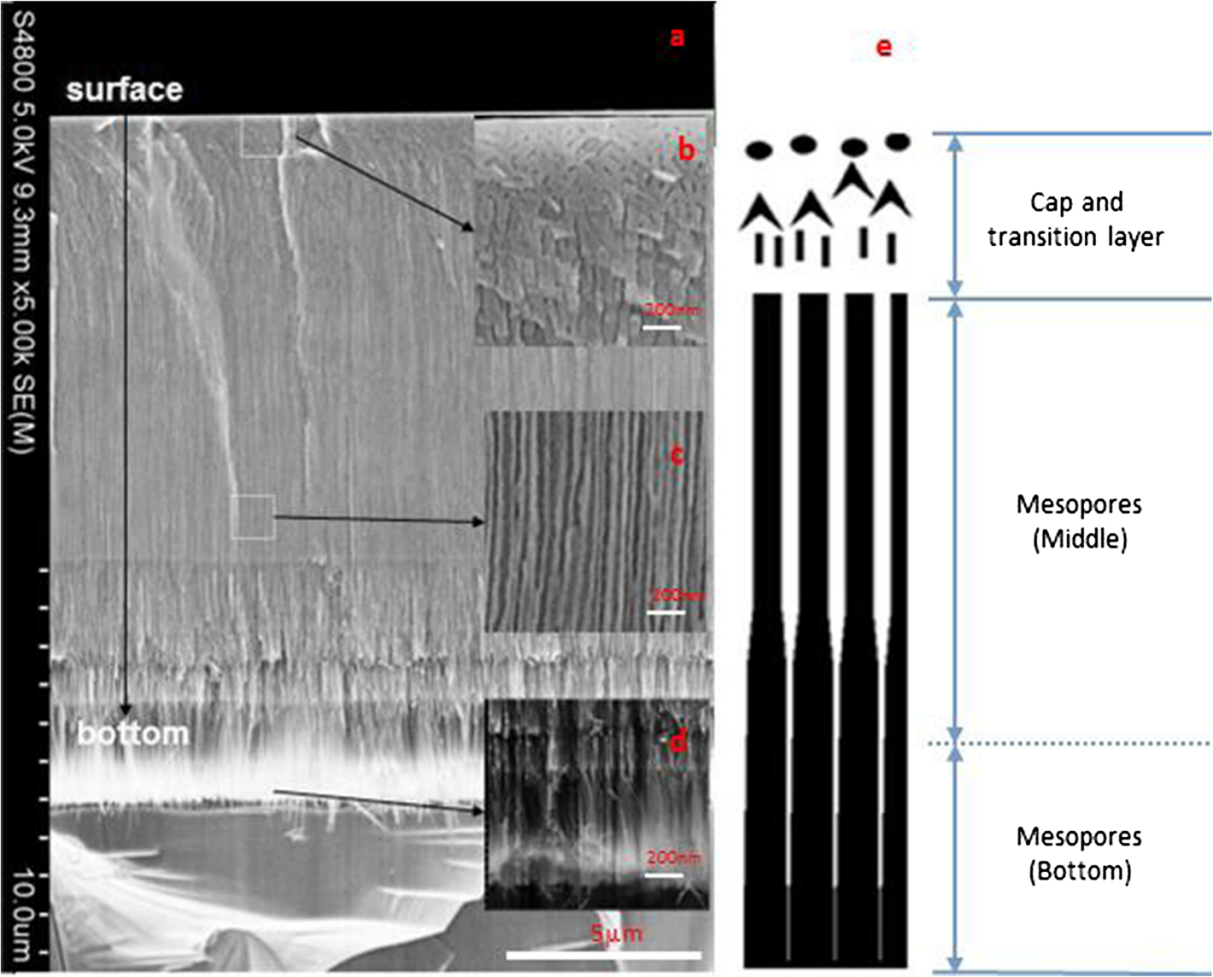
**The cross-section morphology of #3.** The whole view **(a)** and the magnified field of the cap and transition layer **(b)**, mesopores in the middle **(c)**, and mesopores in the bottom **(d)**. The schematic of the cross-section structure is shown on the left **(e)**.

Comparing #1 with #3, we note that the pores of #1 looks discontinuous like with a diameter of about 19 nm in the middle and 33 nm in the bottom. On the contrary, the continuous pores, with the enlarging diameter from the surface to the bottom like #1, are formed for sample #3 (Figure 2). In fact, both sample pore distributions are same; the discontinuous-like pore of #1 is just the pore wall belonging to a pore which grew in front of the pore primarily looked at, which could be found in other porous semiconductors. In conclusion, the *T* from 0.2 to 10 ms cannot influence the pores morphology obviously.

**Figure 3:**
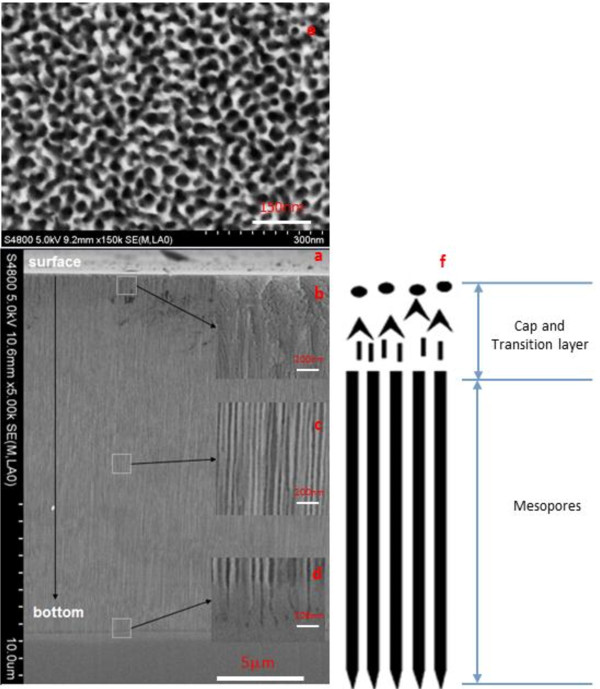
**The cross-section morphology of #4.** The whole view **(a)** and the magnified field of cap and transition layer **(b)**, mesopores in the middle **(c),** and mesopores in the bottom **(d)**. The plane view **(e)** at 1-μm deep is realized by RIE and SEM. The schematic cross-section structure **(f)**.

The samples with *T*_off_ have uniform longitudinal pore distribution. The diameters in the middle and the bottom are the same with reference to #2, #4, and #6. The diameters are enlarged in the bottom of the other samples (#1, #3, #5, #7) without *T*_off_. The cross-section morphology of #3 and #4 are chosen as examples to clarify the role of *T*_off_, as shown in Figures 2 and 3.

When the constant voltage is applied to #7, the columnar mesopores also forms, but the longitudinal view of them is not uniform which also confirms the role of *T*_off_ in slowing down the tendency of pore diameter enlargement. In all etching processes, voltage was increased with the time going due to the increasing specific surface area.

Comparing #6 with #4 and #5 with #3, we can figure out almost the same morphologies in two groups. The conclusion is that the Ohmic contact plays the same role as the Schottky contact in our experiments, which should attribute to high-contact resistance of the Ohmic contact which is close to the Schottky contact in the 10- to 20-V region.

The effect of H_2_O_2_ is expected to facilitate the uniform etching process similar to the Si system [9]. What is more, HF/H_2_O_2_ does have an effect on enhancing uniformity on 6H-SiC [10]. The electrochemical etching SiC process in other works, without H_2_O_2_, can be listed as the following [11]:

(1) $$\mathrm{SiC}+4H_{2}O+8H^{+}\to {SiO}_{2}+{CO}_{2}↑+8H^{+}$$

(2) $$\mathrm{SiC}+2H_{2}O+4H^{+}\to SiO+CO↑+4H^{+}$$

(3) $${SiO}_{2}+6HF\to {\left({SiF}_{6}\right)}^{2-}+2H_{2}O+2H^{+}$$

(4) $$SiO+6HF\to {\left({SiF}_{6}\right)}^{2-}+H_{2}O+4H^{+}$$

When H_2_O_2_ is added to the etching solution, the possible reaction will occur.

(5) $$\mathrm{SiC}+2H_{2}O+4H^{+}+2O^{-}\to {SiO}_{2}+{CO}_{2}↑+4H^{+}$$

The Equation (5) enhances the oxide formation rate. The kinetic balance between the oxide formation and dissolution can therefore be quickly achieved, which favors the formation of columnar pores. The last but not the least, the effect of H_2_O_2_ here aims to promise the pore uniformity in the plane perpendicular to the etching direction.

The mechanism of forming longitudinal uniform mesopores can be stated as the following, which emphasizes the role of *T*_off_ in the etching process. If etching goes without *T*_off_, the diffusion limitation of the reactive species due to the increasing aspect ratio of the pores results in a depletion of the reactive species at the pore tips; thus, a reduced etching rate and accumulation of holes at the pore tips will widen the pore tips. This is the reason why the pore diameter became larger with deeper etching as reported by Gautier et al. [7]. If etching goes with *T*_off_, the concentration of the holes at the pore tips will be decreased, the oxide formation velocity is therefore reduced. However, the reactive species diffusing from the etching solution to pore tips continues. The oxide removal velocity is unchangeable in this case, which prevents pore diameter from being enlarged. With different *T* or *T*_off_ in same current density, the pore diameter and the pore densities are almost the same, which means they would not influence the thickness of pore walls in our experiment.

Additionally, the constant voltage experiment results in inhomogeneous pore structure, because the electric field distribution is not homogenous on the SiC surface.

## Conclusions

In this paper, the effect of cycle time and pause time on the mesopore morphology is clarified. Cycle time ranging from 0.2 to 10 ms does not distinguish pore morphologies, and the different metal contact types has no influence on changing the morphologies either, but the pause time of the constant pulsed current does make a difference on preventing the pore diameters being wider at the bottom.

## Abbreviations

RIE: reactive ion etching; SEM: scanning electron microscope.

## Competing interests

The authors declare that they have no competing interests.

## Authors’ contributions

JHT wrote the manuscript and performed the porous SiC. HH participated in the study of the porous SiC fabrication. YC, YJS, and GJZ offered the critical parameters to make the Ohmic contact on the samples in the experiment. YHL performed RIE on the sample. ZZC read and approved the current manuscript. All authors read and approved the final manuscript.
